# Environmental cues and genes involved in establishment of the superinfective Pf4 phage of *Pseudomonas aeruginosa*

**DOI:** 10.3389/fmicb.2014.00654

**Published:** 2014-12-02

**Authors:** Janice G. K. Hui, Anne Mai-Prochnow, Staffan Kjelleberg, Diane McDougald, Scott A. Rice

**Affiliations:** ^1^The Centre for Marine Bio-Innovation and the School of Biotechnology and Biomolecular Sciences, The University of New South WalesSydney, NSW, Australia; ^2^The Singapore Centre on Environmental Life Sciences Engineering and The School of Biological Sciences, Nanyang Technological UniversitySingapore, Republic of Singapore

**Keywords:** *Pseudomonas aeruginosa*, Inovirus, filamentous phage, superinfection, biofilm, variants, oxidative stress

## Abstract

Biofilm development in *Pseudomonas aeruginosa* is in part dependent on a filamentous phage, Pf4, which contributes to biofilm maturation, cell death, dispersal and variant formation, e.g., small colony variants (SCVs). These biofilm phenotypes correlate with the conversion of the Pf4 phage into a superinfection (SI) variant that reinfects and kills the prophage carrying host, in contrast to other filamentous phage that normally replicate without killing their host. Here we have investigated the physiological cues and genes that may be responsible for this conversion. Flow through biofilms typically developed SI phage approximately days 4 or 5 of development and corresponded with dispersal. Starvation for carbon or nitrogen did not lead to the development of SI phage. In contrast, exposure of the biofilm to nitric oxide, H_2_O_2_ or the DNA damaging agent, mitomycin C, showed a trend of increased numbers of SI phage, suggesting that reactive oxygen or nitrogen species (RONS) played a role in the formation of SI phage. In support of this, mutation of *oxyR*, the major oxidative stress regulator in *P. aeruginosa*, resulted in higher level of and earlier superinfection compared to the wild-type (WT). Similarly, inactivation of *mutS*, a DNA mismatch repair gene, resulted in the early appearance of the SI phage and this was four log higher than the WT. In contrast, loss of *recA*, which is important for DNA repair and the SOS response, also resulted in a delayed and decreased production of SI phage. Treatments or mutations that increased superinfection also correlated with an increase in the production of morphotypic variants. The results suggest that the accumulation of RONS by the biofilm may result in DNA lesions in the Pf4 phage, leading to the formation of SI phage, which subsequently selects for morphotypic variants, such as SCVs.

## INTRODUCTION

It is increasingly accepted that biofilms, or surface-attached communities, account for the majority of bacteria in the environment and that planktonic cells may be more relevant for the dissemination of cells between biofilm habitats (McDougald et al., 2012). This shift in understanding has driven a substantial amount of research focused on understanding how and why bacteria make biofilms and while there are some commonalities, not surprisingly, the regulation of biofilm development is quite complex. Indeed, biofilm formation by *Pseudomonas aeruginosa*, one of the most intensively studied biofilm-forming bacteria, has been suggested to involve around 10% of its genomic potential (Whiteley et al., 2001; Hentzer et al., 2005; Manos et al., 2008). Biofilm development has been shown to be affected by nutrient conditions, and to involve quorum sensing, adhesion proteins, and proteins involved in the turnover of c-di-GMP. Additionally, it has been shown that an endogenous prophage, Pf4, also plays an important role in biofilm development, stress tolerance, dispersal, the formation of morphotypic variants and virulence (Rice et al., 2009).

The Pf4 phage, which has a genome comprised of 12 Kbp, is a member of the Inoviridae and is closely related to ssDNA phage such as M13 and fd. Such phage are continuously secreted by the bacterial host without cell lysis. The effect of the Pf4 phage on biofilm development and variant formation is linked to the establishment of a superinfection (SI) variant of the Pf4 phage. The SI phage are able to form plaques on the wild-type (WT) host, which is otherwise immune to reinfection by the non-SI phage (Rice et al., 2009). Additionally, we have recently shown through deep sequencing of the biofilm dispersal population that the phage accumulates mutations at a significantly higher frequency than the rest of the PAO1 genome, suggesting that superinfection is linked to mutations in the Pf4 prophage (McElroy et al., 2014). Because the formation of the SI phage is an integral part of the biofilm development life-cycle, it is likely that the establishment of the SI phenotype is the result of biofilm-specific physiology and gene expression. However, the specific conditions that lead to SI are currently not known.

Biofilms consist of a stratified population, where cells in different parts of the biofilm exhibit varied physiologies due to differences in nutrient gradients, oxygen gradients, signaling molecules and the accumulation of metabolic products. For example, oxygen gradients can be formed by the failure of oxygen to penetrate through the biofilm as oxygen is respired by the upper layer of cells (Stewart and Franklin, 2008). Similarly, it has been demonstrated that a gradient of nutrients is established, where the high concentration present in the bulk phase is rapidly depleted in the deeper or thicker parts of the biofilm (Zhang et al., 1995; Stewart, 2003). This can result in the reduction of nutrient availability and causes stress to the biofilm cells leading to nutrient starvation in the biofilm’s interior. Conversely, there is a higher concentration of metabolic by-products in the interior of the biofilm compared to the cells in the upper layer of the biofilm, where such waste products may freely diffuse away from the biofilm into the bulk solution.

The accumulation of waste products such as reactive oxygen and nitrogen species (RONS) in biofilms leads to oxidative and nitroxidative stress (Webb et al., 2003; Barraud et al., 2006). For example, aerobic bacteria generate high concentrations of electrons during redox reactions, especially during respiration. These reactions also are partly responsible for the release of different species of oxygen (Cabiscol et al., 2000). This results in the build up of ROS and can lead to DNA damage, protein carbonylation, cofactor degradation, and lipid peroxidation. Therefore, bacteria are dependent on oxidative stress defense systems to mitigate the accumulation of oxygen and oxygen derivatives, e.g., hydrogen peroxide (H_2_O_2_), superoxide anions (O^2-^) and hydroxyl radicals (OH^-^). Bacteria counteract oxidative stress by expressing enzymes to detoxify ROS and repair damage (Storz and Imlay, 1999; Vinckx et al., 2010). This again supports the observation that bacteria in the biofilm interior differ from those on the exterior surface not only in general physiology, but also in terms of gene expression. Therefore, it is anticipated that stress responses play important roles in host defense to constantly changing environments similar to that observed in biofilms. In *P. aeruginosa,* the OxyR transcriptional regulator is the key global regulator of the oxidative stress response, regulating 56 genes (Wei et al., 2012), including *katA, katB, ahpB*, *ahpCF, sodA,* and *sodB*. In addition, it was recently shown that OxyR binds to the Pf4 phage genome (Wei et al., 2012) and, therefore, may play an important role in phage production during conditions of oxidative stress.

DNA damage can be repaired through a number of pathways, including the methyl directed mismatch repair (MMR) and the RecA recombination repair systems. The MMR corrects errors that occur during DNA replication and is a key factor in minimizing mutations during replication (Modrich, 1991). RecA acts as a recombinase and facilitates translesion synthesis during DNA repair as well as facilitating the cleavage of the LexA repressor during the SOS response (Cox, 2007). Interestingly, RecA has been linked to the conversion of lambda phage from lysogeny into lytic replication. Given their role in DNA repair and the observation that the phage accumulates a significant number of mutations at the time of dispersal (McElroy et al., 2014), DNA repair systems may play important roles in the formation of the SI phage.

Here, the physiological triggers that lead to mutations and hence SI and variant formation were determined, and the role of DNA repair systems in formation of the SI Pf4 phage investigated. Multiple inducers were tested, including starvation for different key nutrients, oxidative stress, exposure to H_2_O_2_ and mitomycin C. The induced DNA damage from H_2_O_2_ and mitomycin C resulted in increased occurrence of SI. Similarly, mutational inactivation of the oxidative stress response regulator, OxyR as well as the inactivation of the MMR response via mutation of *mutS* gene, resulted in earlier phage production and a higher titer of the SI Pf4 phage. Interestingly, loss of RecA resulted in a decrease in the formation of SI phage suggesting that recombination may be important in this process.

## MATERIALS AND METHODS

### BACTERIAL STRAINS AND CULTURE CONDITIONS

All *P. aeruginosa* strains (**Table 1**) were cultured in Bertani (1951) medium supplemented with 1% (w/v) NaCl (LB10) or in M9 minimal medium containing: 48 mM NaHPO_4_, 22 mM KH_2_PO_4_, 9 mM NaCl, 19 mM NH_4_Cl, 2 ml MgSO_4_, 100 μM CaCl_2_, supplemented with 15 mM glucose (M9 complete medium). Strains were maintained on LB10 agar (1.5% w/v agar) plates and incubated overnight at 37°C. Liquid cultures were incubated overnight at 37°C with shaking (200 rpm).

**Table 1:** List of *Pseudomonas aeruginosa* strains used in this study.

Strain	Reference
PAO1 WT	Laboratory stock
PAO1 ΔPf4	Rice et al. (2009)
PAO1 SCV2	This study
PAO1 Δ*mutS*	Jacobs et al. (2003)
PAO1 Δ*recA*	Jacobs et al. (2003)
PAO1 Δ*oxyR*	Wei et al. (2012)

### BIOFILM EXPERIMENTS

#### Planktonic cultures

Planktonic cultures were cultivated in 15 ml centrifuge tubes (Falcon) in M9 complete medium and incubated overnight at 37°C with shaking at 200 rpm for 24 h. For stress conditions, 1 mL was collected, centrifuged at 10,000 × *g* for 3 min and the supernatant was discarded. Starvation was induced by replacing M9 complete medium with a solution of M9 salts without glucose (carbon starvation) or without ammonium chloride (nitrogen starvation) as medium for 3 days. Cultures were exposed to nitric oxide (NO) by supplementing the M9 complete medium with the NO donor sodium nitroprusside (SNP; Sigma Aldrich) at 10 μM, 100 μM, and 1 mM for 3 days. Oxidative stress was induced by supplementing M9 complete medium with 100 μM, 1 mM and 10 mM H_2_O_2_ (Biorad) for 3 days. Mitomycin C (Sigma Aldrich) was added to M9 complete medium at 3, 30 and 150 μM to induce DNA damage and cultures were treated for 3 days. All treatments were performed as biological triplicates and were compared to cultures maintained in M9 complete medium. The samples were collected daily to determine the phage titre.

#### Batch biofilms

Batch biofilms were cultivated in tissue culture treated 24 well microtiter plates (Falcon). Briefly, overnight cultures grown in M9 complete medium were diluted 1:100 and 1 ml of the diluted culture was inoculated into each well. Biofilms were formed for 1 d before being treated. Carbon and nitrogen starvation treatments were performed as above and the biofilms were incubated for a further 3 days. Biofilms were exposed to NO, H_2_O_2_ and Mitomycin C as described above and incubated for a further 3 d at 37°C with shaking at 80 rpm. Medium and treatments were replaced daily and at the same time, 1 mL of the biofilm supernatant was harvested to determine plaque forming units (PFUs, see below). All treatments were performed as biological triplicates and were compared to biofilms maintained in M9 complete medium.

#### Continuous-culture biofilm

Biofilms were established in silicone tubing (inner diameter 2.64 ± 0.28 mm and outer diameter 4.88 ± 0.28 mm; Silastic^®^ laboratory tubing). Overnight cultures grown in M9 medium were diluted 1:100 and 2 ml of the diluted culture was inoculated into the tubing using a syringe with a 15G needle, and the bacteria were allowed to attach to the tubing under conditions of no flow for 1 h at room temperature. After the attachment phase, M9 complete medium was pumped through the tubing at a flow rate of 6 ml/h at room temperature. Biofilms were allowed to form for 2 days before treatment. Carbon and nitrogen starvation was induced as above and starvation medium was supplied to the biofilm for 5 days. Similarly, biofilms were exposed to NO by supplementing the M9 complete medium with 1 mM SNP for 5 days. H_2_O_2_ (10 mM) and mitomycin C were added to the M9 complete medium and fed to the biofilm for 5 days. All treatments were performed as biological triplicates and were compared to biofilms maintained in M9 complete medium. The samples were collected daily for CFU and PFU counts.

#### CFU counts and morphological variants from the biofilm effluent

CFUs and the numbers of variants were obtained by collecting 5 ml of the biofilm effluent on days 2–7. Serial dilutions were spread plated onto LB10 agar. The plates were incubated overnight for at least 12 h at 37°C. The plates were incubated for additional 12 h at room temperature to facilitate observation of morphotypic variants.

#### Phage assays

The supernatant was tested for SI phage using a modified version of the top-layer agar method previously described (Eisenstark, 1967). Briefly, bacterial lawns of either the WT PA01 (SI phage) or the Pf4 mutant (total phage) were prepared by mixing 500 μl of an overnight culture, grown in M9 complete medium, with 5 ml of 0.8% (w/v) molten LB10 agar at 55°C. The mixture was poured onto a LB10 agar. The biofilm effluent was centrifuged at 13,000 × *g* for 5 min and filtered through a 0.22 μm filter (Millipore Millex GP) to obtain cell-free supernatant. The supernatant was serially diluted and 10 μl drops were spotted onto the bacterial lawns, air-dried and incubated overnight at 37°C to observe and quantify plaque formation.

## RESULTS

In *P. aeruginosa* PAO1 biofilms, the lysogenic Pf4 prophage converts into its SI form during the dispersal phase and can be detected by the ability of the SI phage to form plaques on lawns of the WT *P. aeruginosa*. This indicates that the SI phage can infect and kill *P. aeruginosa*, which is otherwise resistant to reinfection by the WT Pf4 phage. This is accompanied by the appearance of small colony variants (SCVs) in the dispersal population. To determine the physiological or genetic factors involved in the formation of SI, strains and chemical treatments associated with DNA damage or spontaneous mutation, including *mutS*, *recA*, *oxyR* mutants, H_2_O_2_, mitomycin C, and the NO donor SNP were tested. To facilitate testing a wide range of treatments and conditions, experiments were performed on planktonic and batch biofilms for higher throughput. While the planktonic cultures produced SI phage, their appearance was inconsistent (data not shown) and indeed, the control cultures displayed a random pattern of SI induction. When the same treatments were tested on batch grown biofilms, no SI was observed, even after 3 days of cultivation (data not shown). The control biofilms also showed no SI, suggesting that batch biofilms in microtiter plates, over the 3 days tested, do not replicate flow cell conditions sufficiently to allow for the formation of the SI phage.

**FIGURE 1:**
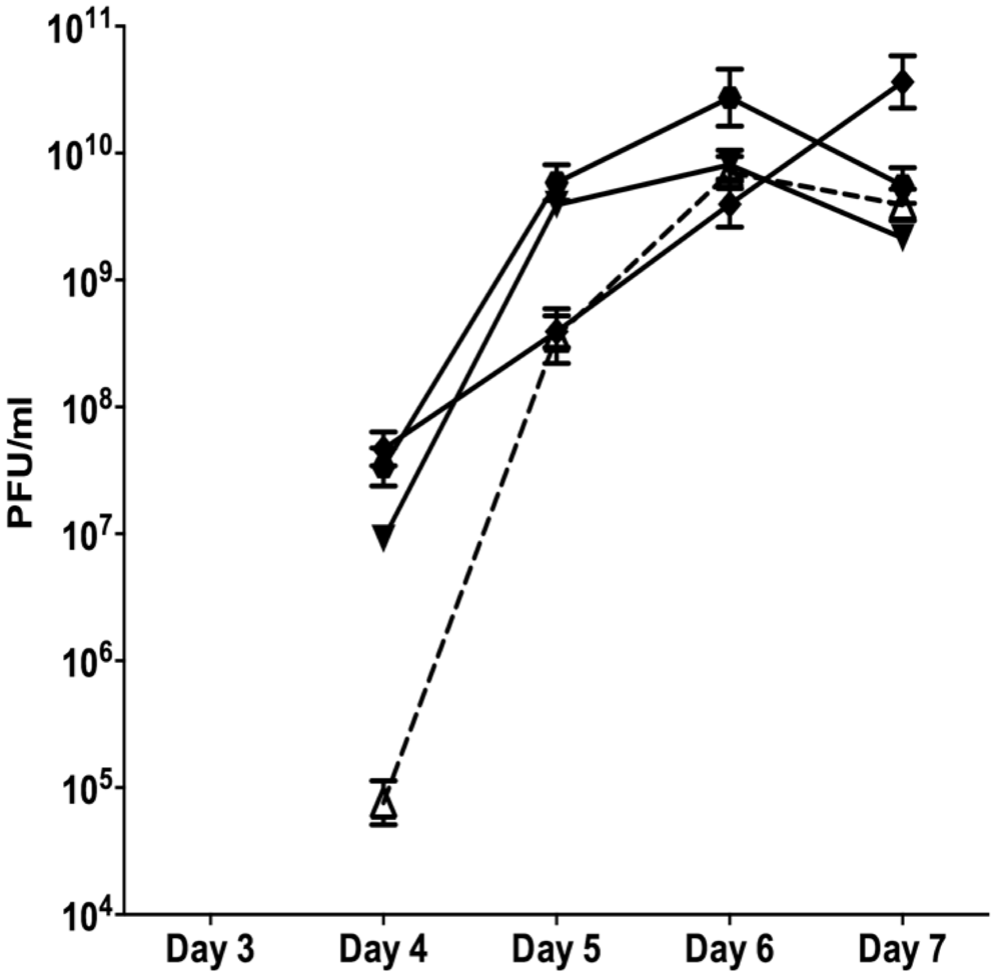
**The appearance of SI Pf4 phage during biofilm development of PAO1 wild-type (WT; open triangle with dotted line), PAO1 treated with mitomycin C (hexagon), PAO1 treated with H_**2**_O_**2**_ (diamond) and PAO1 treated with SNP (inverted triangle).** The SI Pf4 phage was detected via the plaque assay with the PAO1 WT as the target lawn. Data represent the means of three independent experiments and error bars show SE of the mean.

Since neither planktonic nor batch biofilms resulted in reproducible patterns of SI induction, subsequent experiments were conducted in flow through biofilms. The control biofilm produced SI phage on days 4 at 9.7 × 10^4^ PFU/ml (**Figure 1**). The number of SI phage increased exponentially to days 6, reaching a maximum of 7 × 10^9^ PFU/ml before a slight decline on days 7. The nutrient starved (carbon or nitrogen) biofilms were observed to disperse within 24 h of treatment. This was accompanied by a dramatic decrease in the overall phage titre as observed on the Pf4 lawn, most likely due to the loss of biomass through dispersal (data not shown). These results are in agreement with previous work showing that starvation induces biofilm dispersal (Huynh et al., 2012). Therefore, these experiments were not repeated.

When the continuous culture biofilms were exposed to 10 mM of H_2_O_2_ starting from days 2, the SI phage appeared on days 4 at 4.68 × 10^7^ PFU/ml and increased throughout the duration of the experiment reaching a maximum of 3.65 × 10^10^ PFU/ml on days 7. PAO1 biofilms treated with the NO donor SNP, had 9.12 × 10^7^ PFU/ml in the biofilm effluent on days 4. The number of PFU/ml increased on days 5 and 6, reaching a maximum of 8.10 × 10^9^ PFU/ml on days 6 and decreased on days 7 to 2.14 × 10^9^ PFU/ml. This pattern of change in PFU numbers observed from the SNP treated biofilms was also observed when the biofilm was exposed to DNA damaging agent, mitomycin C (**Figure 1**). There was an exponential increase in PFUs starting on days 4 of 3.4 × 10^7^ PFU/ml that reached a plateau on days 6 at 1.9 × 10^10^ PFU/ml and subsequently declined on days 7 to 5.6 × 10^9^ PFU/ml. While the results were not statistically significant (two-way ANOVA), the trends, seen in multiple experiments, suggest that DNA damaging agents or NO exposure may play a role in the formation of SI phage. Further, there was a two-log difference on days 4 for all of the treatments relative to the control, suggesting that these treatments have an effect on the titre of the SI phage even though they did not induce the earlier production of SI phage.

**FIGURE 2:**
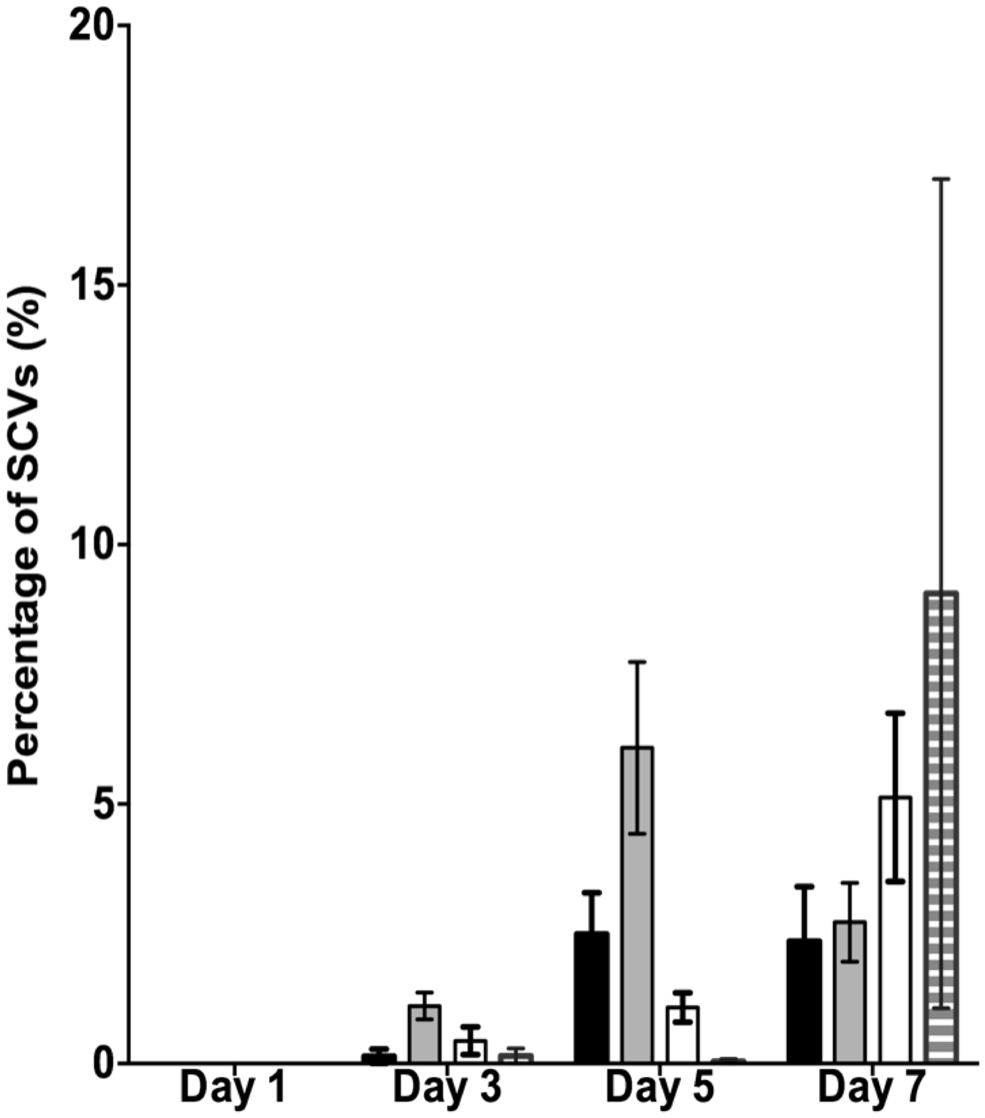
**The percentage of small colony variants (SCVs) from the PAO1 biofilm untreated (black bars), treated with 150 μM of mitomycin C (gray bars), treated with 10 mM of H_**2**_O_**2**_ (white bars) and treated with 1 mM of SNP (striped bars).** Data represent the means and SE of the mean of three experiments.

It has previously been shown that SI Pf4 phage can induce the formation of SCVs (Rice et al., 2009). Therefore, the effluents from the biofilms treated with mitomycin C, H_2_O_2_ and SNP were plated to quantify the number of SCVs relative to the untreated control biofilms (**Figure 2**). The effluent of biofilms exposed to mitomycin C consisted of 1.1% SCVs on days 3 and increased to 6.1% SCVs on days 5. In comparison, the untreated PAO1 biofilm effluent consisted of less than 1% of SCVs on days 3 and 2.5 and 2.4% of SCVs on days 5 and 7, respectively. The DNA damaging agent mitomycin C induced the highest frequency of SCVs over the first 5 days, peaking at 7.5%. In contrast, exposure to H_2_O_2_ and SNP had a lesser effect. For example, there were < 1% SCVs on days 3 from the biofilm exposed to H_2_O_2_, which increased on days 5 and 7, reaching 5.1% SCVs on days 7. Similarly, the biofilm exposed to SNP had less than 1% of SCVs from days 3, reaching its maximum of 9.1% of SCVs on days 7. In comparison to the untreated PAO1 biofilm, there was a two and fourfold increase in SCVs from the biofilm effluent on days 7 for the H_2_O_2_ and SNP treated biofilms, respectively. While the results were not statistically significant when averaged across the three independent experiments (*p* > 0.05, two-way ANOVA), the trend of more SCVs in the treated biofilms relative to the controls was consistent across multiple experiments.

**FIGURE 3:**
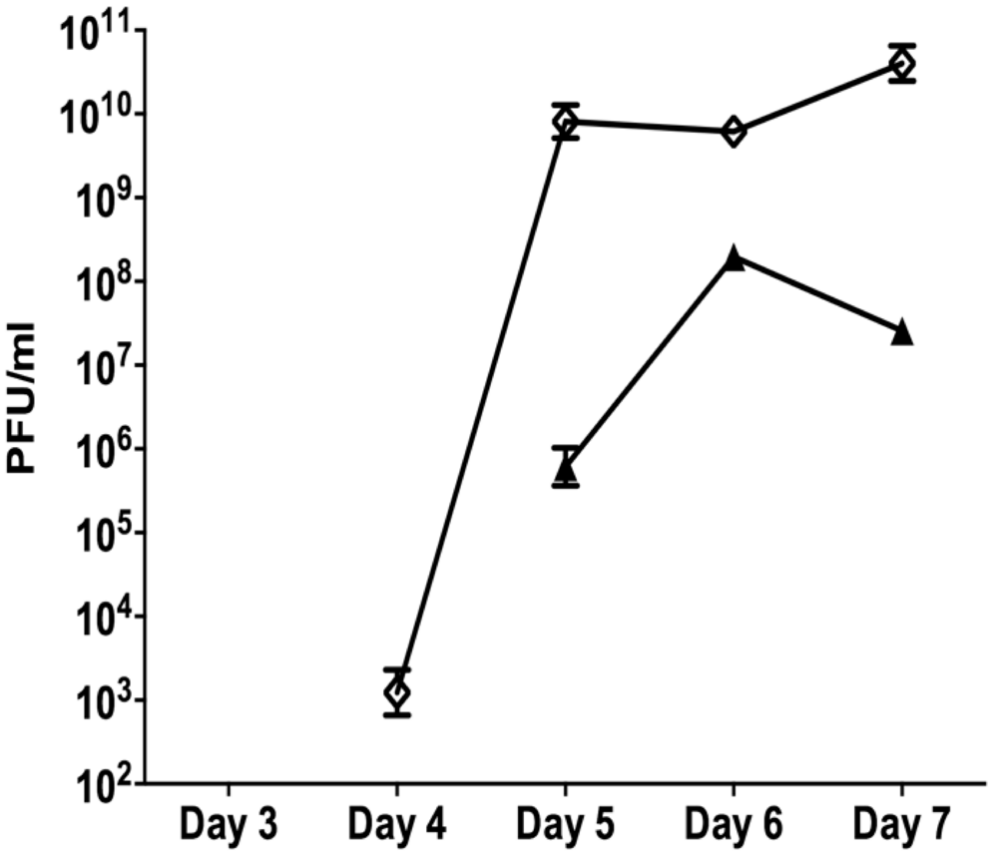
**The role of OxyR in the development of superinfection (SI).** Biofilm effluents from the PAO1 WT (triangle) and *oxyR* mutant (diamond) were screened for the appearance of the SI Pf4 phage, using the soft agar overlay method with the PAO1 WT as the target lawn. Data are the means and SEM of three independent experiments. Note that error bars are present for all data points, but may be too small to visualize on the graph.

**FIGURE 4:**
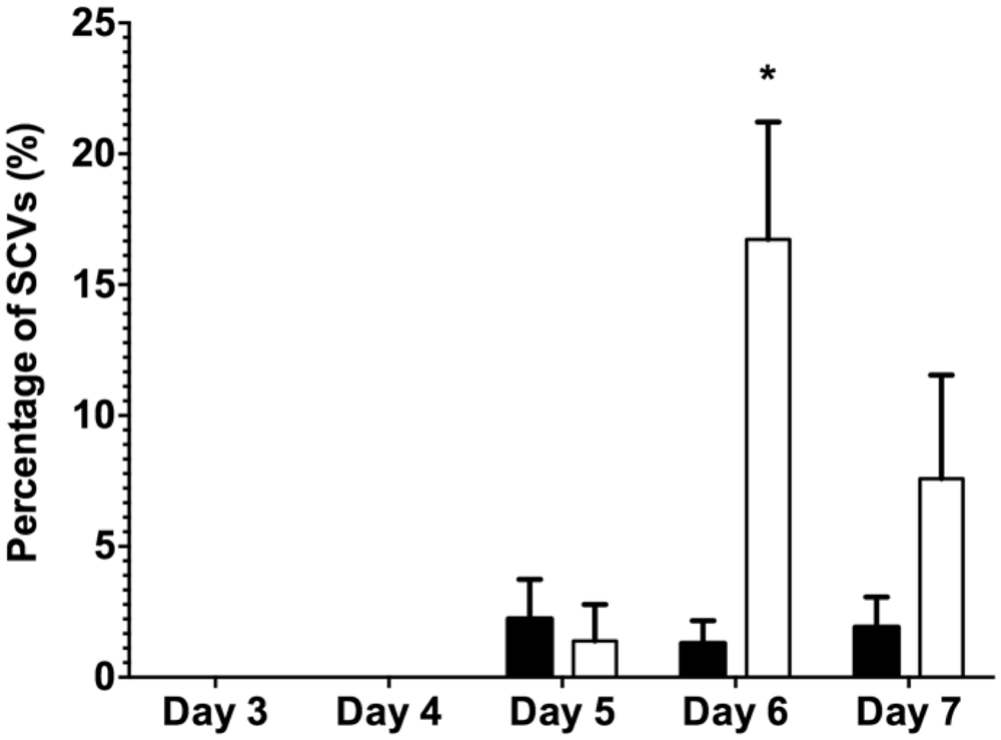
**The percentage of SCVs from the dispersal population of the PAO1 WT biofilm (black bars) and the PAO1 *oxyR* mutant biofilm (white bars).** Colony forming units were determined from biofilm effluents for phenotypic variants from the biofilms. Data represent the means of three experiments and error bars show SE of the mean. (*) indicates a statistically significant difference when compared to PAO1 WT at 95% confidence interval with two-way ANOVA with a Sidak’s post test.

The data presented above (**Figures 1** and **2**) suggests that oxidative stress (exposure to H_2_O_2_ and SNP) and DNA damage (exposure to mitomycin C) may lead to the development of the SI phage, resulting in the appearance of SCVs. Oxidative stress in *P. aeruginosa* is primarily controlled by the transcriptional regulator, OxyR (Storz and Imlay, 1999). This protein changes in conformation upon exposure to oxidative stress, allowing it to bind to specific promoters to control their expression. Therefore, to investigate the role of the global transcriptional regulator, OxyR, in the conversion of the Pf4 phage into its SI form during biofilm development, the *oxyR* mutant biofilm was compared with the PAO1 WT biofilm. The *oxyR* mutant biofilm showed early conversion of the SI Pf4 phage on days 4 as compared to the PAO1 WT biofilm, which produced SI phage on days 5 in these experiments (**Figure 3**). On days 4 for the *oxyR* mutant biofilm, there were 1.2 × 10^3^ PFU/ml detected in the biofilm effluent, and the number increased to 8.1 × 10^9^ PFU/ml on days 5. The *oxyR* mutant biofilm was also observed to have a higher percentage of SCVs during the late stages of biofilm development (**Figure 4**). The PAO1 WT biofilm generated 1.3% SCVs on days 6 and 1.9% on days 7 in comparison to 16.7% and 7.6% of SCVs from the *oxyR* mutant biofilm on days 6 and 7, respectively. The difference between the two biofilms was statistically significant on days 6, with more than 10-fold difference in SCVs. The same trend was observed in the three independent experiments; therefore it is highly likely that OxyR plays an important role in the conversion to the SI and the appearance of SCVs.

**FIGURE 5:**
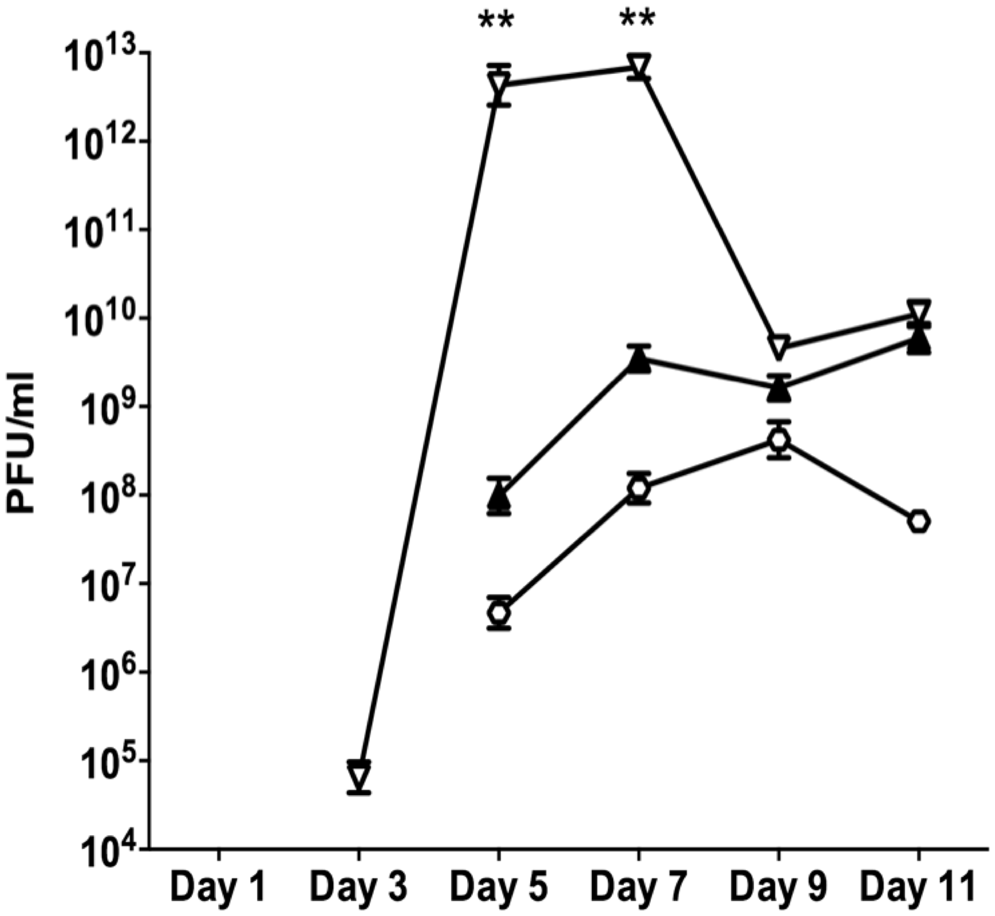
**The appearance of the SI Pf4 phage during biofilm development for PAO1 (closed triangle), the *mutS* mutant (inverted triangle) and the *recA* mutant (hexagon).** Data are the means of three independent experiments and the error bars show SE of the mean. (**) indicates a statistically significant difference compared to PAO1 WT at the 99% confidence interval as determined using a one-way ANOVA.

The early appearance and increased number of SI phage observed for the *oxyR* mutant further supports the possibility that oxidative stress and DNA damage are associated with this conversion. Bacteria have evolved a number of mechanisms to repair damaged DNA and two of the most important mechanisms are the MMR system and the RecA recombination system. To mimic the loss of a functional repair system, a *mutS* mutant biofilm was compared to the PAO1 WT biofilm. The *mutS* mutant biofilm produced 6.5 × 10^4^ PFU/ml of SI phage on days 3 compared to the PAO1 WT biofilm where conversion did not occur until days 5 (**Figure 5**). Additionally, on days 5 the *mutS* mutant produced significantly (*P* < 0.01) more phage (4.3 × 10^12^ PFU/ml) than the WT (9.8 × 10^7^ PFU/ml). The number of PFU from the *mutS* mutant biofilm decreased to 4.6 × 10^9^ PFU/ml on days 9 and 11. In contrast, the *recA* mutant showed a substantial reduction in the number of SI phage (4.7 × 10^6^ PFU/ml) compared to the PAO1 WT biofilm, with Pf4 phage conversion occurring on days 5 of biofilm development (**Figure 5**). Indeed, the number of SI phage observed in the biofilm effluent for the *recA* mutant was approximately one log lower than the WT at all time points tested. This suggests that loss of repair increases the appearance of SI and that the formation of SI phage may also be partially dependent on RecA-mediated recombination activity.

**FIGURE 6:**
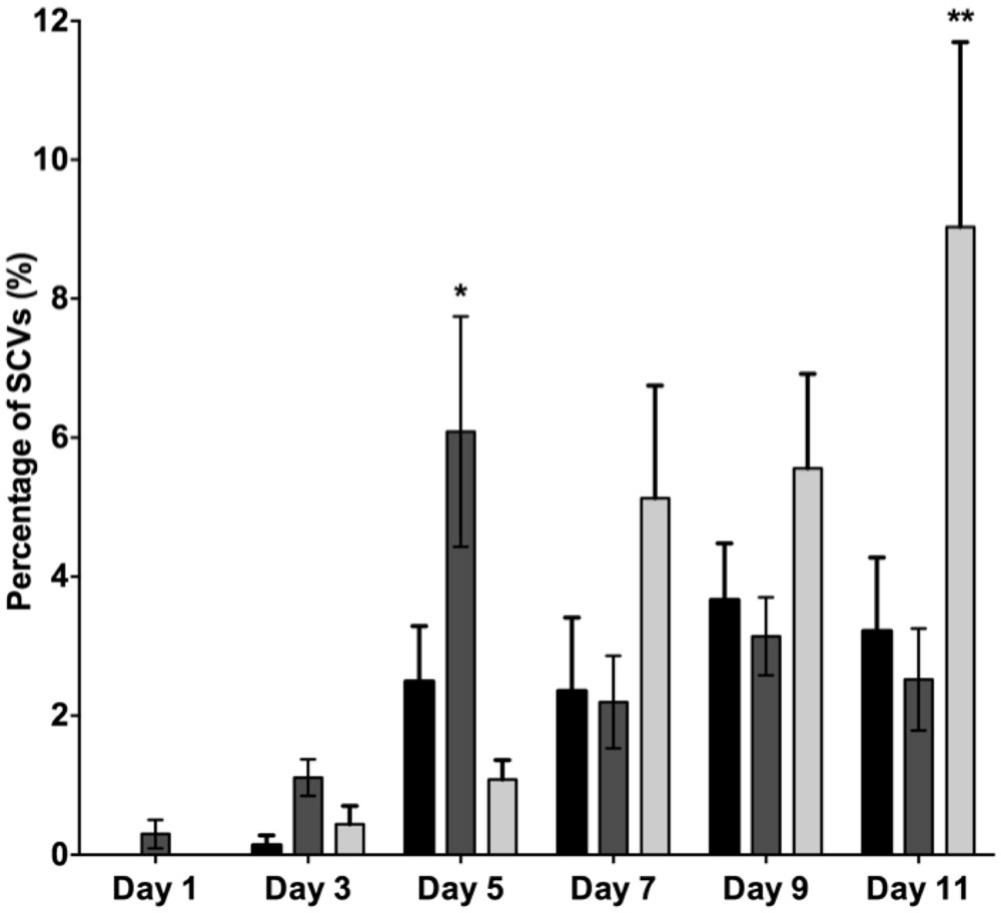
**The percentage of SCVs in the biofilm dispersal population for PAO1 WT (black bars), the *mutS* mutant (dark gray) and the *recA* mutant (light gray).** Data are means of three experiments and the error bars indicate SE of the mean. (*) and (**) indicate a statistically significant difference when compared to PAO1 WT at 95 and 99% confidence intervals respectively, as determined by using a one-way ANOVA.

When comparing the dispersal variants from the biofilms, SCVs were observed from the *mutS* mutant biofilm from days 1 onward and peaked on days 5 at 6.1% SCVs, which was statistically significantly different (*P* < 0.05) to the WT biofilm with 2.5% of SCVs on days 5 (**Figure 6**). While the number of SCV’s was higher for the *mutS* mutant on days 1–5, the percentage of SCVs produced by the *mutS* mutant was similar to the WT from days 7 onward. In contrast, the percentage of SCVs observed in the effluent of the *recA* mutant biofilm was not statistically different from the WT for days 1–3. However, from days 7 onward, the *recA* mutant generated significantly more SCVs than the WT and the *mutS* mutant. For example, on days 11 the *recA* mutant produced 9% SCVs, representing a statistically significant difference (*P* < 0.01) compared to the WT at 3.2% SCVs. These data suggest that the appearance of the SI phage correlates with the onset of detection of the SCV’s in biofilm effluent but does not correlate with the titre of the SI phage. For example, the *recA* mutant produced less SI phage, but significantly more SCVs than either the WT or the *mutS* mutant (**Figure 6**), which produced >four log more phage than the *recA* mutant from days 7 to 11 (**Figure 5**).

## DISCUSSION

It was previously shown that growth in a biofilm leads to the formation of a SI variant of the Pf4 phage (Rice et al., 2009). While biofilm dispersal has previously been linked to nutrient starvation and the accumulation of NO (Sauer et al., 2004; Barraud et al., 2006; Huynh et al., 2012), the specific metabolic or stress conditions that lead to the establishment of the SI phage has not been determined. The results presented here strongly suggest that SI conversion is linked to a dysfunctional oxidative stress response and the MMR system. This is supported by the results showing that chemical treatments that are linked with either DNA damage (mitomycin C) or oxidative stress (H_2_O_2_ and SNP) also result in increased or early development of SI. Oxidative stress is a consequence of the build up of RONS as a result of endogenous by-products that accumulate during aerobic metabolism and/or upon external exposure to ROS, such as the oxidative burst of immune cells. Aerobic bacteria naturally generate high concentrations of electrons and multiple oxygen species through oxidative phosphorylation and respiration. This results in the build up of ROS such as H_2_O_2_, O^2-^ and hydroxyl radical (OH^-^; Henle and Linn, 1997; Storz and Imlay, 1999). It has been shown that ROS and RONS accumulate within microcolonies of the biofilm leading to cell death (Barraud et al., 2006) and that microcolonies within the biofilms are hot spots for the accumulation of mutants (Conibear et al., 2009). While bacterial cells are constantly exposed to intracellularly generated ROS, they are also exposed to exogeneous ROS generated by other microorganisms which can either be used to eliminate competitors or can be released by immune cells to kill invading pathogens (Klotz and Hutcheson, 1992; Brunder et al., 1996). For example, the release of superoxides by the phagocyte NADPH oxidase is a host defense mechanism used by macrophages against pathogens (Miller and Britigan, 1997; Babior, 1999; Janssen et al., 2003).

Hydrogen peroxide can freely diffuse across cellular membranes, making it a lethal antimicrobial as it causes DNA damage through strand breakage (Ananthaswamy and Eisenstark, 1977), deoxynucleotide base damage (Rhaese and Freese, 1968), deoxynucleotide base release (Ward and Kuo, 1976) and DNA cross-linking (Massie et al., 1972). It has previously been shown that the rate of damage caused by OH^-^, the product of H_2_O_2_ degradation via the Fenton reaction, is greater than damage caused by H_2_O_2_, as OH^-^ have a greater ability to bind to DNA (Ananthaswamy and Eisenstark, 1977; Ward et al., 1987). The fact that OH^-^ are more damaging, could explain the observation that H_2_O_2_ had a relatively minor effect on the biofilm compared to the loss of the OxyR regulator (*oxyR* mutant biofilm), which acts in response to combinations of RONS. Exposure to H_2_O_2_ gives rise to oxidative stress, however, bacterial cells harbor repair mechanisms that repair DNA damage and produce enzymes to scavenge and remove H_2_O_2_ (Ma and Eaton, 1992). Therefore, H_2_O_2_ may induce the expression of SI phage by a few hours because it has limited capacity to damage the cell in comparison to the loss of OxyR. In the case of the OxyR mutant, the SI phage was induced a day earlier than in the PAO1 WT biofilm which may reflect the broader role of OxyR in controlling the global oxidative stress response.

It was determined that OxyR of *P. aeruginosa* binds to multiple sites in the genome (Wei et al., 2012). Interestingly, one of those sites is the intergenic region between PA0716 and PA0717 genes of the Pf4 genome. The binding region lies within the ORF of the repressor *c* gene of the prophage genome. This suggests that interactions of the OxyR protein with the Pf4 phage genome are important in SI conversion and overall control of phage production. One possible mechanism is that the OxyR normally binds to the repressor C promoter and represses gene expression. When the repressor C acquires mutations, these may prevent binding of the OxyR to the phage genome leading to overproduction of the phage particles. Binding assays of the OxyR to the repressor *c* gene and/or competition binding between OxyR and repressor C may elucidate the role of OxyR in SI phage conversion and the interactions between the OxyR regulator and the prophage. Deep sequencing data of the *P. aeruginosa* dispersal population and associated phage indicates that there is a high frequency of mutations in the *repC* gene and the upstream promoter region, while there are no other mutations in the phage genome (McElroy et al., 2014).

Inactivation of the MMR system is associated with the highly mutable state, called hypermutation. For example, *P. aeruginosa* CF isolates have been shown to lack the *mutS* and *mutY* genes of the MMR system leading to the hypermutatable phenotype (Oliver et al., 2000). The *mutS* protein plays an important role in recognizing mismatches and initiates the repair by association with other Mut proteins, the loss of this protein will completely arrest the MMR system. Our results suggest that loss of a functional MMR system leads to early conversion of the SI phage and higher SCV numbers. This supports the possibility that mutations in the genome lead to the conversion of the SI phage and that this process is linked to DNA damage via oxidative stress and requires active MMR functions to reduce conversion to the SI phage.

The mutations could arise in either the prophage genome, the replicative form (RF) or in the ssDNA phage genome as it is replicated for packing into phage particles. In the latter two cases, the mutated RF or phage genome (after conversion into dsDNA) could be introduced into the prophage locus via recombination. The loss of RecA leads to a decrease in the SI Pf4 phage in the biofilm, suggesting that RecA is required for the conversion to the SI phage. RecA plays roles in both the induction of the SOS response and in recombination based DNA repair (Schlacher and Goodman, 2007) and it is not clear from the data presented here, which function of RecA is associated with the formation of the SI phage. It is possible that the mutations resulting in SI Pf4 occur during phage replication and that the mutated phage genome is recombined with the genomic prophage, resulting in fixation of the mutation in the bacterial host, although this is yet to be experimentally determined.

*Pseudomonas aeruginosa* biofilms have been shown to generate genetic diversity by producing phenotypic variants with a variety of functions such as the production of pyomelanin for protection against oxidative stress (Boles et al., 2004), loss of flagellar and twitching motility for enhanced adherence to surfaces (Deziel et al., 2001), and increased tolerance against antibiotic treatment (Drenkard and Ausubel, 2002). These adaptive phenotypes are important for the survival and the fitness of bacteria. However, deep sequencing data (McElroy et al., 2014) suggest that there are a limited number of mutations in the host genome outside of the phage region. This could suggest that instead of frequent random mutations, the conversion of the Pf4 phage to its SI form may drive the formation of the phenotypic variants observed from the biofilm through an as yet unknown mechanism. Genetic variation is important for the increased stress and antimicrobial tolerance of *P. aeruginosa* biofilms (Boles et al., 2004) and the formation of SI phage has been shown to increase variant formation. Therefore, the process of SI formation could have negative treatment implications, where SI leads to the increased number of resistant variants during chronic, biofilm related infections.

## CONCLUSION

The conversion into the SI Pf4 phage coincided with the appearance of SCVs from the dispersal population of the PAO1 biofilm. In the work presented here, formation of the SI phage appears to be correlated with a functional MMR system and the oxidative stress response mediated by OxyR. Further, the results presented here suggest that high levels of either DNA damaging agents or ROS can induce the SI phenotypes. Therefore, it is likely that during biofilm maturation, high concentrations of RONS accumulate due to endogenous metabolism, overwhelming the ability of the host to detoxify DNA damaging molecules, leading to the accumulation of mutations. The nucleotide composition of the repressor *c* gene may predispose it to acquire single nucleotide polymorphisms at frequencies higher than the host genome and such mutations disrupt or change the immunity function of the repressor C protein, allowing the mutant phage to subsequently reinfect hosts with WT immunity functions. This would lead to strong selection pressure in the biofilm for variants that are resistant to the SI (and additionally carry the SI Pf4 phage) that subsequently persist. The SI phage, through an unknown mechanism, also drives changes in the morphology of the SI host. Therefore, it is of interest to understand how SI results in morphotypic variation, the involvement of the repressor *c* gene and the mechanism by which OxyR controls SI.

## AUTHOR CONTRIBUTIONS

Janice G. K. Hui designed and performed experiments, contributed to the interpretation of results, wrote the manuscript and approved the final version for publication; Anne Mai-Prochnow, Staffan Kjelleberg, Diane McDougald, and Scott A. Rice designed experiments, contributed to the interpretation of results, wrote the manuscript and approved the final version for publication.

## Conflict of Interest Statement

The authors declare that the research was conducted in the absence of any commercial or financial relationships that could be construed as a potential conflict of interest.
